# Bis(4-carbamoylpiperidinium) biphenyl-4,4′-disulfonate

**DOI:** 10.1107/S1600536810045526

**Published:** 2010-11-13

**Authors:** Graham Smith, Urs D. Wermuth, David J. Young

**Affiliations:** a Faculty of Science and Technology, Queensland University of Technology, GPO Box 2434, Brisbane, Queensland 4001, Australia; b Faculty of Science, Universiti Brunei Darussalam, Jalan Tungku Link BE 1410, Negara Brunei Darussalam

## Abstract

In the title isonipecotamide salt 2C_6_H_13_N_2_O^+^·C_12_H_8_O_6_S_2_^2−^, the asymmetric unit comprises one biphenyl-4,4′-disulfonate dianion which lies across a crystallographic inversion centre and another in a general position [dihedral angle between the two phenyl rings is 37.1 (1)°], together with three isonipecotamide cations. Two of these cations give a cyclic homomeric amide–amide dimer inter­action [graph set *R*_2_^2^(8)], the other giving a similar dimeric inter­action but across an inversion centre, both dimers then forming lateral cyclic *R*_4_^2^(8) pyrimidinium–amide N—H⋯O inter­actions. These units are linked both laterally and longitudinally to the sulfonate groups of the dianions through piperidinium N—H⋯O hydrogen bonds, giving a three-dimensional framework structure.

## Related literature

For structural data on bipyridine-4,4′-disulfonate salts and related compounds, see: Swift & Ward (1998 ▶); Swift *et al.* (1998 ▶); Holman & Ward (2000 ▶); Liao *et al.* (2001 ▶); Smith *et al.* (2010 ▶). For isonipecotamide salt structures, see: Smith & Wermuth (2010*a* ▶,*b* ▶,*c* ▶). For graph-set motifs, see: Etter *et al.* (1990 ▶).
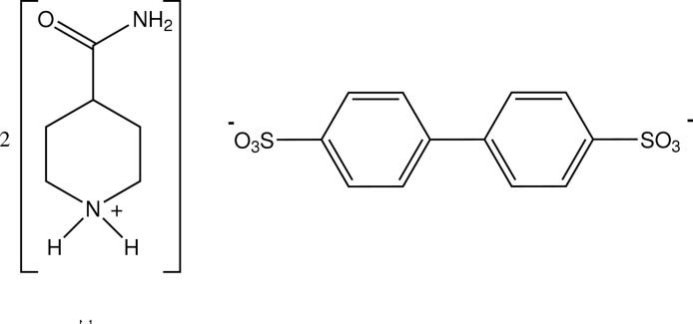


## Experimental

### 

#### Crystal data

2C_6_H_13_N_2_O^+^·C_12_H_8_O_6_S_2_^−^
*M* *_r_* = 570.69
Triclinic, 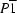

*a* = 8.2530 (4) Å
*b* = 16.0418 (8) Å
*c* = 16.7408 (11) Å
α = 112.255 (5)°
β = 97.166 (5)°
γ = 101.714 (4)°
*V* = 1958.2 (2) Å^3^
*Z* = 3
Mo *K*α radiation
μ = 0.26 mm^−1^
*T* = 200 K
0.40 × 0.40 × 0.20 mm

#### Data collection

Oxford Diffraction Gemini-S Ultra CCD-detector diffractometer
Absorption correction: multi-scan (*CrysAlis PRO*; Oxford Diffraction, 2009 ▶) *T*_min_ = 0.911, *T*_max_ = 0.980
23474 measured reflections
7679 independent reflections
6364 reflections with *I* > 2σ(*I*)
*R*_int_ = 0.031

#### Refinement

*R*[*F* ^2^ > 2σ(*F* ^2^)] = 0.035
*wR*(*F* ^2^) = 0.098
*S* = 1.09
7679 reflections
562 parameters
H atoms treated by a mixture of independent and constrained refinement
Δρ_max_ = 0.40 e Å^−3^
Δρ_min_ = −0.51 e Å^−3^

### 

Data collection: *CrysAlis PRO* (Oxford Diffraction, 2009 ▶); cell refinement: *CrysAlis PRO*; data reduction: *CrysAlis PRO*; program(s) used to solve structure: *SHELXS97* (Sheldrick, 2008 ▶); program(s) used to refine structure: *SHELXL97* (Sheldrick, 2008 ▶) within *WinGX* (Farrugia, 1999 ▶); molecular graphics: *PLATON* (Spek, 2009 ▶); software used to prepare material for publication: *PLATON*.

## Supplementary Material

Crystal structure: contains datablocks global, I. DOI: 10.1107/S1600536810045526/fl2328sup1.cif

Structure factors: contains datablocks I. DOI: 10.1107/S1600536810045526/fl2328Isup2.hkl

Additional supplementary materials:  crystallographic information; 3D view; checkCIF report

## Figures and Tables

**Table 1:** Hydrogen-bond geometry (Å, °)

*D*—H⋯*A*	*D*—H	H⋯*A*	*D*⋯*A*	*D*—H⋯*A*
N1*C*—H11*C*⋯O42*A*^i^	0.91 (2)	1.99 (2)	2.889 (2)	169 (2)
N1*C*—H12*C*⋯O45*A*^ii^	0.90 (3)	1.95 (3)	2.838 (2)	168.1 (18)
N1*D*—H11*D*⋯O43*A*	0.90 (2)	2.04 (2)	2.878 (2)	154.6 (16)
N1*D*—H11*D*⋯O43*B*	0.90 (2)	2.407 (18)	2.855 (2)	111.0 (15)
N1*D*—H12*D*⋯O46*A*^iii^	0.91 (2)	1.98 (2)	2.877 (2)	170 (2)
N1*E*—H11*E*⋯O42*B*	0.901 (19)	2.003 (19)	2.878 (2)	163.5 (18)
N1*E*—H12*E*⋯O44*A*^iv^	0.93 (2)	2.508 (18)	2.908 (2)	106.2 (15)
N41*C*—H42*C*⋯O41*E*	0.89 (3)	1.95 (3)	2.836 (3)	178 (2)
N41*D*—H41*D*⋯O41*D*^v^	0.94 (3)	2.00 (3)	2.936 (3)	171 (2)
N41*D*—H42*D*⋯O41*C*^vi^	0.85 (3)	2.34 (3)	3.134 (2)	158 (2)
N41*E*—H41*E*⋯O41*D*^vii^	0.89 (3)	2.16 (3)	2.996 (2)	158 (3)
N41*E*—H42*E*⋯O41*C*	0.89 (3)	2.13 (3)	3.014 (3)	172 (2)

Symmetry codes: (i) 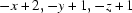; (ii) 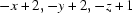; (iii) 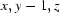; (iv) 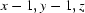; (v) 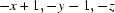; (vi) 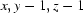; (vii) 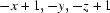.

**Fig. 1.**
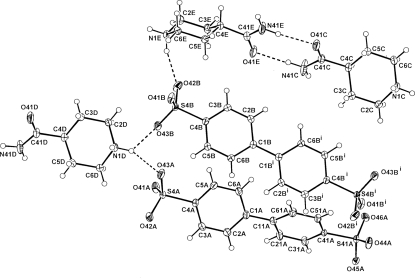
Molecular configuration and atom naming scheme for the three INIPA cations (C, D, E) and the two BPDS dianions (A and B) in the asymmetric unit of (I). The dianion B lies across an inversion centre [for symmetry code (i), see Table 1] and displacement ellipsoids are drawn at the 50% probability level.

**Fig. 2.**
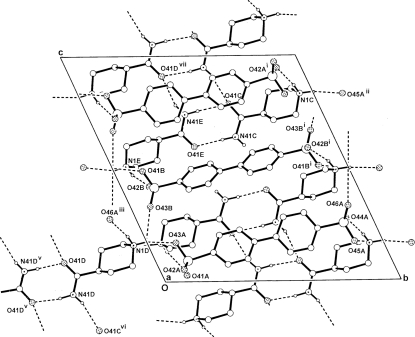
The three-dimensional hydrogen-bonded framework structure of (I) viewed down the a cell direction showing the cyclic R22(8) and R24(8) amide–amide hydrogen-bonding interactions and their extension through the BPDS dianions. Non-associative H atoms are omitted. For symmetry codes, see Table 1.

## supplementary crystallographic information

### Comment

The structures of the Lewis base salts of biphenyl-4,4'-disulfonic acid (BPDS) are not prevalent in the CSD, *e.g.* with β-alanine (Liao *et al.*, 2001) and with 2-(2,4-dinitrobenzyl)pyridine (Smith *et al.*, 2010), but the bis(guanidinium) salt is notable as a co-host structure for cooperative guest recognition in clathrate formation, with numerous aromatic monocyclic and polycyclic hydrocarbons (Swift & Ward, 1998; Swift *et al.*, 1998; Holman & Ward, 2000). The amide 4-carbamoylpiperidine (isonipecotamide, INIPA) is a compound for which there were no structures in the crystallographic literature. We therefore initiated a project aimed at synthesizing a series of salts of INIPA with a number of carboxylic acids, mainly aromatic, with a view of producing crystalline materials suitable for X-ray structural analysis. This amide has proved to be a particularly useful synthon for this purpose, giving the structures of largely anhydrous 1:1 salts with picric acid, 3,5-dinitrosalicylic acid (two polymorphs) (Smith & Wermuth, 2010*a*) as well as with the three isomeric mononitrobenzoic acids and 3,5-dinitrobenzoic acid (Smith & Wermuth, 2010*b*). All of these are 1:1 anhydrous salts while the acetate (Smith & Wermuth, 2010*c*) is a monohydrate.

Our reaction of 4-carbamoylpiperidine with biphenyl-4,4'-disulfonic acid in aqueous ethanol gave good anhydrous crystals of the title compound, (I) and the structure is reported here. With compound (I) (Fig. 1), the asymmetric unit comprises one BPDS dianion (*B*) which lies across a crystallographic inversion centre and another dianion (*A* in a general position, together with three INIPA anions (*C, D, E*). Two of these anions (*C* and *E*) give a cyclic dimeric amide–amide interaction [graph set *R*^2^_2_(8) (Etter *et al.*, 1990)], the other giving a similar but monomeric interaction across an inversion centre. The two dimers also give a lateral cyclic *R*^2^_4_(8) amide-amide interaction (Table 1), these units being linked both laterally and longitudinally to the sulfonate groups of the dianions through piperidinium N—H···O hydrogen bonds, giving a three-dimensional framework structure (Fig 2). One of the amide H-atoms (H41*C*) has no possible H-bond association.

With all three isonipecotamide cations the amide group is rotated *ca* 100° out of the plane of the benzene ring [comparative torsion angles C3/C5–C4–C41–O41: 107.02 (19)° (*C*), 108.90 (19)° (*D*), 100.16 (19)° (*E*]. In the planar BPDS *B* dianions there are short intramolecular H2B···H6B^i^/H6B···H2B^i^ contacts (2.07 Å) [for symmetry code (i) see Table 1] similar to those observed in the structure of the 2-(2,4-dinitrobenzyl)pyridinium salt of BPDS (Smith *et al.*, 2010), in which the dianion is also centrosymmetric. The two phenyl rings of the *A* dianions are non-coplanar [torsion angle C2*A*–C1*A*–C11*A*–C21*A*, 143.92 (19)°].

### Experimental

The title compound was synthesized by heating together under reflux for 10 minutes, 2 mmol of 4-carbamoylpiperidine (isonipecotamide) and 1 mmol of biphenyl-4,4'-disulfonic acid in 50 ml of 50% ethanol–water. After concentration to *ca* 30 ml, partial room temperature evaporation of the hot-filtered solution gave large colourless plates of (I) from which a specimen was cleaved for the X-ray analysis.

### Refinement

Hydrogen atoms involved in hydrogen-bonding interactions were located by difference methods and their positional and isotropic displacement parameters were refined. The H-atoms were included in the refinement at calculated positions [C–H = 0.93 Å (aromatic) or 0.97 Å (aliphatic)] and with *U*_iso_(H) = 1.2*U*_eq_(C), while using a riding-model approximation.

### Crystal data

**Table:**

2C_6_H_13_N_2_O^+^·C_12_H_8_O_6_S_2_^−^	*Z* = 3
*M**_r_* = 570.69	*F*(000) = 906
Triclinic, *P*1	*D*_x_ = 1.452 Mg m^−^^3^
Hall symbol: -P 1	Mo *K*α radiation, λ = 0.71073 Å
*a* = 8.2530 (4) Å	Cell parameters from 8867 reflections
*b* = 16.0418 (8) Å	θ = 3.2–28.8°
*c* = 16.7408 (11) Å	µ = 0.26 mm^−^^1^
α = 112.255 (5)°	*T* = 200 K
β = 97.166 (5)°	Plate, colourless
γ = 101.714 (4)°	0.40 × 0.40 × 0.20 mm
*V* = 1958.2 (2) Å^3^	

### Data collection

**Table:**

Oxford Diffraction Gemini-S Ultra CCD-detector diffractometer	7679 independent reflections
Radiation source: Enhance (Mo) X-ray source	6364 reflections with *I* > 2σ(*I*)
graphite	*R*_int_ = 0.031
ω scans	θ_max_ = 26.0°, θ_min_ = 3.2°
Absorption correction: multi-scan (*CrysAlis PRO*; Oxford Diffraction, 2009)	*h* = −10→10
*T*_min_ = 0.911, *T*_max_ = 0.980	*k* = −19→19
23474 measured reflections	*l* = −20→20

### Refinement

**Table:**

Refinement on *F*^2^	Primary atom site location: structure-invariant direct methods
Least-squares matrix: full	Secondary atom site location: difference Fourier map
*R*[*F*^2^ > 2σ(*F*^2^)] = 0.035	Hydrogen site location: inferred from neighbouring sites
*wR*(*F*^2^) = 0.098	H atoms treated by a mixture of independent and constrained refinement
*S* = 1.09	*w* = 1/[σ^2^(*F*_o_^2^) + (0.0573*P*)^2^ + 0.215*P*] where *P* = (*F*_o_^2^ + 2*F*_c_^2^)/3
7679 reflections	(Δ/σ)_max_ = 0.002
562 parameters	Δρ_max_ = 0.40 e Å^−^^3^
0 restraints	Δρ_min_ = −0.51 e Å^−^^3^

### Special details

**Table:**

Geometry. Bond distances, angles *etc*. have been calculated using the rounded fractional coordinates. All su's are estimated from the variances of the (full) variance-covariance matrix. The cell e.s.d.'s are taken into account in the estimation of distances, angles and torsion angles
Refinement. Refinement of *F*^2^ against ALL reflections. The weighted *R*-factor *wR* and goodness of fit *S* are based on *F*^2^, conventional *R*-factors *R* are based on *F*, with *F* set to zero for negative *F*^2^. The threshold expression of *F*^2^ > σ(*F*^2^) is used only for calculating *R*-factors(gt) *etc*. and is not relevant to the choice of reflections for refinement. *R*-factors based on *F*^2^ are statistically about twice as large as those based on *F*, and *R*- factors based on ALL data will be even larger.

### Fractional atomic coordinates and isotropic or equivalent isotropic displacement parameters (Å^2^)

**Table:**

	*x*	*y*	*z*	*U*_iso_*/*U*_eq_	
S4A	0.82703 (5)	0.13767 (3)	0.09291 (3)	0.0190 (1)	
S41A	1.06493 (5)	0.86976 (3)	0.25435 (3)	0.0176 (1)	
O41A	0.66717 (15)	0.10391 (8)	0.02904 (8)	0.0316 (4)	
O42A	0.96363 (15)	0.10371 (8)	0.05433 (9)	0.0298 (4)	
O43A	0.81159 (17)	0.12200 (8)	0.17254 (8)	0.0320 (4)	
O44A	1.20766 (16)	0.92224 (8)	0.32924 (8)	0.0325 (4)	
O45A	1.09354 (15)	0.87918 (8)	0.17312 (8)	0.0262 (4)	
O46A	0.90432 (15)	0.88868 (8)	0.27438 (8)	0.0274 (4)	
C1A	0.9569 (2)	0.45553 (11)	0.17383 (11)	0.0232 (5)	
C2A	1.0003 (3)	0.39525 (13)	0.09888 (13)	0.0360 (6)	
C3A	0.9652 (3)	0.29980 (13)	0.07588 (13)	0.0328 (6)	
C4A	0.88737 (19)	0.26213 (11)	0.12834 (10)	0.0187 (5)	
C5A	0.8481 (2)	0.32059 (11)	0.20427 (11)	0.0253 (5)	
C6A	0.8821 (2)	0.41650 (12)	0.22664 (11)	0.0264 (5)	
C11A	0.9856 (2)	0.55740 (11)	0.19442 (11)	0.0228 (5)	
C21A	0.9620 (3)	0.58684 (12)	0.12643 (12)	0.0317 (6)	
C31A	0.9889 (2)	0.68127 (12)	0.14384 (11)	0.0278 (5)	
C41A	1.03869 (19)	0.74839 (10)	0.23078 (10)	0.0179 (4)	
C51A	1.0628 (2)	0.72092 (11)	0.29954 (11)	0.0222 (5)	
C61A	1.0355 (2)	0.62611 (12)	0.28137 (11)	0.0254 (5)	
S4B	0.68682 (5)	0.13983 (3)	0.41642 (3)	0.0197 (1)	
O41B	0.76007 (16)	0.12258 (8)	0.49054 (8)	0.0296 (4)	
O42B	0.50633 (15)	0.13283 (9)	0.41138 (9)	0.0337 (4)	
O43B	0.7264 (2)	0.08532 (9)	0.33419 (9)	0.0468 (5)	
C1B	0.9546 (2)	0.44969 (10)	0.48803 (10)	0.0204 (5)	
C2B	0.8136 (2)	0.42503 (12)	0.52040 (12)	0.0295 (6)	
C3B	0.7291 (2)	0.33169 (12)	0.49779 (12)	0.0297 (6)	
C4B	0.7862 (2)	0.26027 (11)	0.44236 (10)	0.0189 (5)	
C5B	0.9239 (3)	0.28263 (13)	0.40861 (15)	0.0405 (7)	
C6B	1.0080 (3)	0.37604 (13)	0.43145 (15)	0.0445 (7)	
O41C	0.57028 (18)	0.62179 (8)	0.78553 (9)	0.0366 (4)	
N1C	0.81523 (19)	0.94193 (10)	0.83011 (10)	0.0240 (4)	
N41C	0.5061 (3)	0.58553 (13)	0.64025 (13)	0.0569 (8)	
C2C	0.8147 (2)	0.88438 (12)	0.73590 (11)	0.0253 (5)	
C3C	0.7609 (2)	0.77985 (11)	0.71370 (11)	0.0241 (5)	
C4C	0.5934 (2)	0.75021 (11)	0.74070 (11)	0.0222 (5)	
C5C	0.6059 (2)	0.81317 (11)	0.83729 (11)	0.0250 (5)	
C6C	0.6456 (2)	0.91528 (12)	0.85156 (12)	0.0268 (5)	
C41C	0.5546 (2)	0.64687 (12)	0.72451 (12)	0.0261 (5)	
O41D	0.4858 (2)	−0.39280 (9)	0.08881 (9)	0.0455 (5)	
N1D	0.68074 (18)	−0.06252 (10)	0.16379 (9)	0.0194 (4)	
N41D	0.4860 (3)	−0.41732 (12)	−0.05238 (12)	0.0491 (7)	
C2D	0.5026 (2)	−0.10376 (11)	0.16829 (11)	0.0217 (5)	
C3D	0.4735 (2)	−0.20812 (11)	0.14410 (11)	0.0236 (5)	
C4D	0.5034 (2)	−0.25961 (11)	0.05190 (11)	0.0215 (5)	
C5D	0.6816 (2)	−0.21258 (11)	0.04612 (11)	0.0225 (5)	
C6D	0.7099 (2)	−0.10792 (11)	0.07259 (11)	0.0221 (5)	
C41D	0.4893 (2)	−0.36272 (12)	0.03098 (12)	0.0284 (5)	
O41E	0.40936 (19)	0.39100 (9)	0.59760 (9)	0.0419 (5)	
N1E	0.30389 (18)	0.06166 (10)	0.51160 (10)	0.0218 (4)	
N41E	0.4681 (3)	0.41519 (13)	0.74075 (12)	0.0486 (7)	
C2E	0.1532 (2)	0.10154 (11)	0.50968 (12)	0.0253 (5)	
C3E	0.2165 (2)	0.20717 (11)	0.53852 (12)	0.0260 (5)	
C4E	0.3342 (2)	0.25597 (11)	0.63063 (11)	0.0235 (5)	
C5E	0.4817 (2)	0.21023 (11)	0.63280 (11)	0.0217 (5)	
C6E	0.4159 (2)	0.10470 (11)	0.60222 (11)	0.0226 (5)	
C41E	0.4060 (2)	0.36065 (12)	0.65494 (12)	0.0279 (6)	
H2A	1.05390	0.41990	0.06390	0.0430*	
H3A	0.99360	0.26100	0.02550	0.0390*	
H5A	0.79910	0.29580	0.24040	0.0300*	
H6A	0.85450	0.45500	0.27750	0.0320*	
H21A	0.92760	0.54240	0.06810	0.0380*	
H31A	0.97360	0.69930	0.09740	0.0330*	
H51A	1.09720	0.76570	0.35780	0.0270*	
H61A	1.05070	0.60830	0.32800	0.0300*	
H2B	0.77440	0.47210	0.55830	0.0350*	
H3B	0.63440	0.31750	0.52000	0.0360*	
H5B	0.96150	0.23510	0.37020	0.0490*	
H6B	1.10180	0.38960	0.40840	0.0530*	
H4C	0.50110	0.75830	0.70390	0.0270*	
H11C	0.896 (3)	0.9334 (14)	0.8666 (14)	0.035 (5)*	
H12C	0.841 (3)	1.0017 (15)	0.8368 (13)	0.040 (6)*	
H21C	0.73720	0.89820	0.69720	0.0300*	
H22C	0.92740	0.90080	0.72550	0.0300*	
H31C	0.74690	0.74480	0.65050	0.0290*	
H32C	0.85010	0.76410	0.74390	0.0290*	
H41C	0.493 (3)	0.6046 (19)	0.6029 (18)	0.065 (9)*	
H42C	0.476 (3)	0.5247 (18)	0.6280 (15)	0.052 (7)*	
H51C	0.69450	0.80460	0.87490	0.0300*	
H52C	0.49940	0.79590	0.85360	0.0300*	
H61C	0.64880	0.95470	0.91260	0.0320*	
H62C	0.55820	0.92410	0.81360	0.0320*	
H4D	0.41820	−0.25600	0.00790	0.0260*	
H11D	0.700 (2)	−0.0010 (14)	0.1780 (12)	0.027 (5)*	
H12D	0.758 (3)	−0.0705 (13)	0.2020 (13)	0.030 (5)*	
H21D	0.42150	−0.09430	0.12760	0.0260*	
H22D	0.48600	−0.07260	0.22760	0.0260*	
H31D	0.35810	−0.23460	0.14580	0.0280*	
H32D	0.54980	−0.21690	0.18740	0.0280*	
H41D	0.484 (3)	−0.4805 (18)	−0.0685 (16)	0.055 (7)*	
H42D	0.494 (3)	−0.3950 (17)	−0.0905 (16)	0.057 (7)*	
H51D	0.76660	−0.22230	0.08460	0.0270*	
H52D	0.69560	−0.24200	−0.01400	0.0270*	
H61D	0.82510	−0.08030	0.07130	0.0270*	
H62D	0.63250	−0.09770	0.03090	0.0270*	
H4E	0.26970	0.24880	0.67400	0.0280*	
H11E	0.364 (2)	0.0718 (12)	0.4732 (12)	0.020 (5)*	
H12E	0.266 (2)	−0.0024 (15)	0.4951 (12)	0.032 (5)*	
H21E	0.08570	0.08870	0.54930	0.0300*	
H22E	0.08230	0.07240	0.45030	0.0300*	
H31E	0.27690	0.21920	0.49610	0.0310*	
H32E	0.12000	0.23310	0.53880	0.0310*	
H41E	0.460 (3)	0.3938 (18)	0.7821 (18)	0.068 (8)*	
H42E	0.508 (3)	0.4760 (17)	0.7557 (15)	0.051 (7)*	
H51E	0.55330	0.22300	0.59470	0.0260*	
H52E	0.55040	0.23760	0.69260	0.0260*	
H61E	0.51080	0.07760	0.60160	0.0270*	
H62E	0.35260	0.09170	0.64320	0.0270*	

### Atomic displacement parameters (Å^2^)

**Table:**

	*U*^11^	*U*^22^	*U*^33^	*U*^12^	*U*^13^	*U*^23^
S4A	0.0211 (2)	0.0127 (2)	0.0207 (2)	0.0049 (2)	0.0017 (2)	0.0052 (2)
S41A	0.0192 (2)	0.0124 (2)	0.0199 (2)	0.0052 (2)	0.0027 (2)	0.0055 (2)
O41A	0.0267 (7)	0.0239 (7)	0.0320 (7)	0.0073 (5)	−0.0051 (5)	0.0021 (5)
O42A	0.0309 (7)	0.0219 (6)	0.0413 (8)	0.0150 (5)	0.0121 (6)	0.0128 (6)
O43A	0.0493 (8)	0.0170 (6)	0.0270 (7)	0.0023 (6)	0.0056 (6)	0.0106 (5)
O44A	0.0327 (7)	0.0181 (6)	0.0346 (7)	0.0023 (5)	−0.0101 (6)	0.0060 (5)
O45A	0.0357 (7)	0.0166 (6)	0.0290 (7)	0.0065 (5)	0.0112 (5)	0.0116 (5)
O46A	0.0286 (7)	0.0271 (7)	0.0342 (7)	0.0173 (5)	0.0124 (5)	0.0142 (6)
C1A	0.0283 (9)	0.0170 (8)	0.0249 (9)	0.0079 (7)	0.0060 (7)	0.0084 (7)
C2A	0.0603 (13)	0.0238 (9)	0.0373 (11)	0.0163 (9)	0.0300 (10)	0.0185 (8)
C3A	0.0530 (12)	0.0220 (9)	0.0325 (10)	0.0184 (9)	0.0247 (9)	0.0121 (8)
C4A	0.0190 (8)	0.0145 (8)	0.0214 (8)	0.0051 (6)	0.0017 (6)	0.0067 (6)
C5A	0.0319 (10)	0.0191 (8)	0.0242 (9)	0.0031 (7)	0.0094 (7)	0.0091 (7)
C6A	0.0367 (10)	0.0190 (9)	0.0219 (9)	0.0080 (7)	0.0104 (8)	0.0055 (7)
C11A	0.0282 (9)	0.0169 (8)	0.0253 (9)	0.0089 (7)	0.0082 (7)	0.0087 (7)
C21A	0.0575 (13)	0.0173 (9)	0.0186 (9)	0.0126 (8)	0.0079 (8)	0.0046 (7)
C31A	0.0461 (11)	0.0207 (9)	0.0201 (8)	0.0135 (8)	0.0092 (8)	0.0095 (7)
C41A	0.0169 (8)	0.0132 (7)	0.0230 (8)	0.0055 (6)	0.0049 (6)	0.0061 (6)
C51A	0.0260 (9)	0.0174 (8)	0.0191 (8)	0.0038 (7)	0.0014 (7)	0.0053 (7)
C61A	0.0333 (10)	0.0206 (9)	0.0232 (9)	0.0070 (7)	0.0034 (7)	0.0112 (7)
S4B	0.0249 (2)	0.0130 (2)	0.0166 (2)	0.0002 (2)	0.0045 (2)	0.0037 (2)
O41B	0.0354 (7)	0.0175 (6)	0.0318 (7)	0.0035 (5)	−0.0030 (6)	0.0109 (5)
O42B	0.0215 (6)	0.0284 (7)	0.0480 (8)	−0.0020 (5)	−0.0003 (6)	0.0193 (6)
O43B	0.0765 (11)	0.0197 (7)	0.0295 (7)	−0.0044 (7)	0.0292 (7)	−0.0018 (6)
C1B	0.0217 (8)	0.0163 (9)	0.0213 (8)	0.0044 (7)	0.0034 (7)	0.0067 (7)
C2B	0.0372 (10)	0.0176 (9)	0.0342 (10)	0.0094 (8)	0.0197 (8)	0.0066 (8)
C3B	0.0333 (10)	0.0204 (9)	0.0365 (10)	0.0053 (8)	0.0201 (8)	0.0103 (8)
C4B	0.0221 (8)	0.0150 (8)	0.0171 (8)	0.0021 (6)	0.0017 (6)	0.0063 (6)
C5B	0.0443 (12)	0.0163 (9)	0.0570 (13)	0.0063 (8)	0.0335 (10)	0.0054 (9)
C6B	0.0456 (12)	0.0184 (9)	0.0676 (15)	0.0040 (9)	0.0410 (11)	0.0097 (9)
O41C	0.0556 (9)	0.0188 (6)	0.0341 (7)	0.0073 (6)	0.0081 (6)	0.0116 (6)
N1C	0.0262 (8)	0.0150 (7)	0.0277 (8)	0.0031 (6)	−0.0010 (6)	0.0091 (6)
N41C	0.1053 (19)	0.0143 (9)	0.0331 (10)	0.0013 (10)	−0.0090 (11)	0.0059 (8)
C2C	0.0263 (9)	0.0234 (9)	0.0280 (9)	0.0057 (7)	0.0068 (7)	0.0128 (8)
C3C	0.0273 (9)	0.0194 (9)	0.0237 (9)	0.0074 (7)	0.0065 (7)	0.0061 (7)
C4C	0.0227 (8)	0.0159 (8)	0.0252 (9)	0.0049 (7)	0.0009 (7)	0.0070 (7)
C5C	0.0276 (9)	0.0192 (9)	0.0283 (9)	0.0070 (7)	0.0106 (7)	0.0084 (7)
C6C	0.0306 (10)	0.0185 (9)	0.0292 (9)	0.0096 (7)	0.0082 (8)	0.0057 (7)
C41C	0.0246 (9)	0.0184 (9)	0.0303 (10)	0.0041 (7)	0.0039 (7)	0.0065 (7)
O41D	0.0883 (12)	0.0197 (7)	0.0329 (8)	0.0176 (7)	0.0187 (8)	0.0127 (6)
N1D	0.0213 (7)	0.0128 (7)	0.0204 (7)	0.0038 (6)	0.0017 (6)	0.0044 (6)
N41D	0.1010 (17)	0.0167 (9)	0.0259 (9)	0.0179 (9)	0.0114 (10)	0.0050 (7)
C2D	0.0216 (8)	0.0179 (8)	0.0238 (8)	0.0066 (7)	0.0063 (7)	0.0058 (7)
C3D	0.0235 (9)	0.0179 (8)	0.0289 (9)	0.0046 (7)	0.0080 (7)	0.0091 (7)
C4D	0.0242 (9)	0.0144 (8)	0.0223 (8)	0.0052 (6)	0.0012 (7)	0.0050 (7)
C5D	0.0266 (9)	0.0206 (8)	0.0201 (8)	0.0108 (7)	0.0061 (7)	0.0058 (7)
C6D	0.0236 (9)	0.0201 (8)	0.0224 (8)	0.0055 (7)	0.0070 (7)	0.0082 (7)
C41D	0.0361 (10)	0.0162 (8)	0.0278 (9)	0.0058 (7)	0.0028 (8)	0.0059 (7)
O41E	0.0664 (10)	0.0169 (6)	0.0357 (8)	0.0029 (6)	0.0097 (7)	0.0087 (6)
N1E	0.0237 (8)	0.0137 (7)	0.0252 (8)	0.0040 (6)	0.0054 (6)	0.0058 (6)
N41E	0.0875 (16)	0.0170 (9)	0.0299 (10)	0.0026 (9)	0.0118 (10)	0.0040 (8)
C2E	0.0216 (9)	0.0196 (9)	0.0308 (9)	0.0053 (7)	0.0022 (7)	0.0077 (7)
C3E	0.0242 (9)	0.0191 (9)	0.0329 (10)	0.0081 (7)	0.0030 (7)	0.0088 (7)
C4E	0.0258 (9)	0.0156 (8)	0.0279 (9)	0.0062 (7)	0.0098 (7)	0.0063 (7)
C5E	0.0196 (8)	0.0208 (9)	0.0214 (8)	0.0033 (7)	0.0044 (7)	0.0064 (7)
C6E	0.0241 (9)	0.0210 (8)	0.0243 (9)	0.0091 (7)	0.0053 (7)	0.0098 (7)
C41E	0.0336 (10)	0.0170 (9)	0.0301 (10)	0.0067 (7)	0.0100 (8)	0.0059 (7)

### Geometric parameters (Å, °)

**Table:**

S4A—O41A	1.4479 (13)	C51A—H51A	0.9300
S4A—O42A	1.4661 (14)	C61A—H61A	0.9300
S4A—O43A	1.4628 (14)	C1B—C2B	1.392 (2)
S4A—C4A	1.7921 (19)	C1B—C6B	1.398 (3)
S41A—O44A	1.4483 (13)	C1B—C1B^i^	1.507 (2)
S41A—O45A	1.4660 (13)	C2B—C3B	1.397 (3)
S41A—O46A	1.4693 (13)	C3B—C4B	1.385 (3)
S41A—C41A	1.7941 (18)	C4B—C5B	1.374 (3)
S4B—O42B	1.4602 (14)	C5B—C6B	1.397 (3)
S4B—O43B	1.4432 (15)	C2B—H2B	0.9300
S4B—C4B	1.7962 (19)	C3B—H3B	0.9300
S4B—O41B	1.4569 (14)	C5B—H5B	0.9300
O41C—C41C	1.234 (2)	C6B—H6B	0.9300
O41D—C41D	1.235 (2)	C2C—C3C	1.527 (3)
O41E—C41E	1.231 (2)	C3C—C4C	1.547 (2)
N1C—C2C	1.495 (2)	C4C—C5C	1.525 (2)
N1C—C6C	1.507 (2)	C4C—C41C	1.532 (3)
N41C—C41C	1.329 (3)	C5C—C6C	1.521 (3)
N1C—H12C	0.90 (3)	C2C—H22C	0.9700
N1C—H11C	0.91 (2)	C2C—H21C	0.9700
N41C—H42C	0.89 (3)	C3C—H32C	0.9700
N41C—H41C	0.80 (3)	C3C—H31C	0.9700
N1D—C2D	1.508 (2)	C4C—H4C	0.9800
N1D—C6D	1.497 (2)	C5C—H52C	0.9700
N41D—C41D	1.331 (3)	C5C—H51C	0.9700
N1D—H11D	0.90 (2)	C6C—H62C	0.9700
N1D—H12D	0.91 (2)	C6C—H61C	0.9700
N41D—H41D	0.94 (3)	C2D—C3D	1.524 (3)
N41D—H42D	0.85 (3)	C3D—C4D	1.529 (2)
N1E—C2E	1.512 (2)	C4D—C5D	1.543 (2)
N1E—C6E	1.493 (2)	C4D—C41D	1.531 (3)
N41E—C41E	1.332 (3)	C5D—C6D	1.524 (3)
N1E—H11E	0.901 (19)	C2D—H22D	0.9700
N1E—H12E	0.93 (2)	C2D—H21D	0.9700
N41E—H42E	0.89 (3)	C3D—H31D	0.9700
N41E—H41E	0.89 (3)	C3D—H32D	0.9700
C1A—C11A	1.497 (3)	C4D—H4D	0.9800
C1A—C6A	1.398 (3)	C5D—H52D	0.9700
C1A—C2A	1.404 (3)	C5D—H51D	0.9700
C2A—C3A	1.386 (3)	C6D—H62D	0.9700
C3A—C4A	1.391 (3)	C6D—H61D	0.9700
C4A—C5A	1.387 (2)	C2E—C3E	1.528 (3)
C5A—C6A	1.396 (3)	C3E—C4E	1.529 (2)
C11A—C21A	1.395 (3)	C4E—C41E	1.535 (3)
C11A—C61A	1.398 (2)	C4E—C5E	1.547 (2)
C21A—C31A	1.394 (3)	C5E—C6E	1.524 (3)
C31A—C41A	1.390 (2)	C2E—H21E	0.9700
C41A—C51A	1.386 (2)	C2E—H22E	0.9700
C51A—C61A	1.396 (3)	C3E—H31E	0.9700
C2A—H2A	0.9300	C3E—H32E	0.9700
C3A—H3A	0.9300	C4E—H4E	0.9800
C5A—H5A	0.9300	C5E—H51E	0.9700
C6A—H6A	0.9300	C5E—H52E	0.9700
C21A—H21A	0.9300	C6E—H61E	0.9700
C31A—H31A	0.9300	C6E—H62E	0.9700
			
O41A—S4A—O42A	113.10 (8)	N1C—C2C—C3C	111.39 (15)
O41A—S4A—O43A	113.03 (8)	C2C—C3C—C4C	112.68 (14)
O41A—S4A—C4A	105.39 (8)	C3C—C4C—C41C	110.21 (14)
O42A—S4A—O43A	112.15 (8)	C3C—C4C—C5C	109.64 (14)
O42A—S4A—C4A	106.45 (8)	C5C—C4C—C41C	111.56 (15)
O43A—S4A—C4A	106.00 (8)	C4C—C5C—C6C	110.43 (15)
O44A—S41A—O45A	113.64 (8)	N1C—C6C—C5C	108.74 (14)
O44A—S41A—O46A	112.80 (8)	O41C—C41C—C4C	122.36 (16)
O44A—S41A—C41A	106.64 (8)	N41C—C41C—C4C	115.82 (17)
O45A—S41A—O46A	111.65 (8)	O41C—C41C—N41C	121.8 (2)
O45A—S41A—C41A	105.75 (8)	N1C—C2C—H21C	109.00
O46A—S41A—C41A	105.63 (8)	H21C—C2C—H22C	108.00
O41B—S4B—C4B	105.82 (8)	C3C—C2C—H21C	109.00
O42B—S4B—O43B	114.33 (9)	C3C—C2C—H22C	109.00
O42B—S4B—C4B	105.54 (9)	N1C—C2C—H22C	109.00
O43B—S4B—C4B	106.30 (8)	C2C—C3C—H31C	109.00
O41B—S4B—O42B	111.02 (8)	H31C—C3C—H32C	108.00
O41B—S4B—O43B	113.05 (9)	C4C—C3C—H31C	109.00
C2C—N1C—C6C	111.72 (14)	C2C—C3C—H32C	109.00
C6C—N1C—H12C	110.7 (16)	C4C—C3C—H32C	109.00
C2C—N1C—H12C	105.9 (13)	C41C—C4C—H4C	108.00
H11C—N1C—H12C	110 (2)	C3C—C4C—H4C	108.00
C2C—N1C—H11C	109.3 (14)	C5C—C4C—H4C	108.00
C6C—N1C—H11C	108.7 (15)	C4C—C5C—H51C	110.00
C41C—N41C—H42C	118.7 (15)	C6C—C5C—H51C	110.00
H41C—N41C—H42C	122 (3)	C6C—C5C—H52C	110.00
C41C—N41C—H41C	119 (2)	H51C—C5C—H52C	108.00
C2D—N1D—C6D	111.19 (13)	C4C—C5C—H52C	110.00
H11D—N1D—H12D	108.3 (18)	H61C—C6C—H62C	108.00
C6D—N1D—H12D	108.2 (14)	C5C—C6C—H62C	110.00
C2D—N1D—H11D	110.7 (11)	C5C—C6C—H61C	110.00
C6D—N1D—H11D	107.7 (12)	N1C—C6C—H62C	110.00
C2D—N1D—H12D	110.6 (15)	N1C—C6C—H61C	110.00
C41D—N41D—H41D	120.1 (15)	N1D—C2D—C3D	109.63 (14)
C41D—N41D—H42D	120.8 (18)	C2D—C3D—C4D	111.47 (15)
H41D—N41D—H42D	119 (2)	C3D—C4D—C41D	111.38 (15)
C2E—N1E—C6E	111.37 (14)	C3D—C4D—C5D	109.69 (14)
C2E—N1E—H12E	109.5 (11)	C5D—C4D—C41D	109.04 (14)
C6E—N1E—H11E	109.5 (11)	C4D—C5D—C6D	112.15 (14)
H11E—N1E—H12E	109.0 (17)	N1D—C6D—C5D	109.64 (14)
C6E—N1E—H12E	107.9 (12)	O41D—C41D—C4D	121.79 (16)
C2E—N1E—H11E	109.5 (12)	O41D—C41D—N41D	122.00 (19)
C41E—N41E—H42E	116.8 (15)	N41D—C41D—C4D	116.18 (17)
H41E—N41E—H42E	120 (2)	N1D—C2D—H22D	110.00
C41E—N41E—H41E	122.5 (19)	N1D—C2D—H21D	110.00
C2A—C1A—C6A	117.65 (18)	C3D—C2D—H22D	110.00
C6A—C1A—C11A	121.55 (15)	H21D—C2D—H22D	108.00
C2A—C1A—C11A	120.78 (17)	C3D—C2D—H21D	110.00
C1A—C2A—C3A	121.5 (2)	H31D—C3D—H32D	108.00
C2A—C3A—C4A	119.86 (19)	C2D—C3D—H31D	109.00
S4A—C4A—C5A	120.85 (14)	C2D—C3D—H32D	109.00
C3A—C4A—C5A	119.74 (18)	C4D—C3D—H32D	109.00
S4A—C4A—C3A	119.29 (13)	C4D—C3D—H31D	109.00
C4A—C5A—C6A	120.16 (16)	C41D—C4D—H4D	109.00
C1A—C6A—C5A	121.03 (16)	C3D—C4D—H4D	109.00
C1A—C11A—C61A	121.88 (16)	C5D—C4D—H4D	109.00
C21A—C11A—C61A	117.61 (17)	C6D—C5D—H51D	109.00
C1A—C11A—C21A	120.51 (16)	C4D—C5D—H51D	109.00
C11A—C21A—C31A	121.60 (17)	C4D—C5D—H52D	109.00
C21A—C31A—C41A	119.81 (17)	H51D—C5D—H52D	108.00
C31A—C41A—C51A	119.70 (16)	C6D—C5D—H52D	109.00
S41A—C41A—C31A	120.26 (13)	N1D—C6D—H62D	110.00
S41A—C41A—C51A	120.01 (12)	N1D—C6D—H61D	110.00
C41A—C51A—C61A	120.03 (15)	C5D—C6D—H62D	110.00
C11A—C61A—C51A	121.24 (17)	H61D—C6D—H62D	108.00
C3A—C2A—H2A	119.00	C5D—C6D—H61D	110.00
C1A—C2A—H2A	119.00	N1E—C2E—C3E	109.19 (14)
C4A—C3A—H3A	120.00	C2E—C3E—C4E	111.85 (15)
C2A—C3A—H3A	120.00	C3E—C4E—C41E	110.66 (15)
C6A—C5A—H5A	120.00	C5E—C4E—C41E	109.62 (14)
C4A—C5A—H5A	120.00	C3E—C4E—C5E	109.63 (14)
C5A—C6A—H6A	119.00	C4E—C5E—C6E	111.42 (14)
C1A—C6A—H6A	120.00	N1E—C6E—C5E	110.18 (15)
C31A—C21A—H21A	119.00	O41E—C41E—C4E	121.27 (16)
C11A—C21A—H21A	119.00	N41E—C41E—C4E	116.03 (17)
C41A—C31A—H31A	120.00	O41E—C41E—N41E	122.7 (2)
C21A—C31A—H31A	120.00	N1E—C2E—H21E	110.00
C41A—C51A—H51A	120.00	N1E—C2E—H22E	110.00
C61A—C51A—H51A	120.00	C3E—C2E—H21E	110.00
C51A—C61A—H61A	119.00	C3E—C2E—H22E	110.00
C11A—C61A—H61A	119.00	H21E—C2E—H22E	108.00
C2B—C1B—C6B	116.53 (17)	C2E—C3E—H31E	109.00
C1B^i^—C1B—C6B	121.34 (16)	C2E—C3E—H32E	109.00
C1B^i^—C1B—C2B	122.13 (15)	C4E—C3E—H31E	109.00
C1B—C2B—C3B	122.04 (17)	C4E—C3E—H32E	109.00
C2B—C3B—C4B	120.08 (16)	H31E—C3E—H32E	108.00
S4B—C4B—C5B	119.76 (15)	C3E—C4E—H4E	109.00
C3B—C4B—C5B	119.07 (18)	C5E—C4E—H4E	109.00
S4B—C4B—C3B	121.16 (14)	C41E—C4E—H4E	109.00
C4B—C5B—C6B	120.6 (2)	C4E—C5E—H51E	109.00
C1B—C6B—C5B	121.6 (2)	C4E—C5E—H52E	109.00
C3B—C2B—H2B	119.00	C6E—C5E—H51E	109.00
C1B—C2B—H2B	119.00	C6E—C5E—H52E	109.00
C4B—C3B—H3B	120.00	H51E—C5E—H52E	108.00
C2B—C3B—H3B	120.00	N1E—C6E—H61E	110.00
C6B—C5B—H5B	120.00	N1E—C6E—H62E	110.00
C4B—C5B—H5B	120.00	C5E—C6E—H61E	110.00
C1B—C6B—H6B	119.00	C5E—C6E—H62E	110.00
C5B—C6B—H6B	119.00	H61E—C6E—H62E	108.00
			
O41A—S4A—C4A—C3A	−81.03 (17)	S41A—C41A—C51A—C61A	177.33 (13)
O41A—S4A—C4A—C5A	94.97 (15)	C41A—C51A—C61A—C11A	0.7 (3)
O42A—S4A—C4A—C3A	39.33 (17)	C2B—C1B—C6B—C5B	0.1 (3)
O42A—S4A—C4A—C5A	−144.66 (14)	C2B—C1B—C1B^i^—C6B^i^	0.3 (3)
O43A—S4A—C4A—C3A	158.91 (16)	C6B—C1B—C1B^i^—C6B^i^	−180.00 (19)
O43A—S4A—C4A—C5A	−25.09 (16)	C6B—C1B—C1B^i^—C2B^i^	−0.3 (3)
O44A—S41A—C41A—C31A	−143.79 (14)	C1B^i^—C1B—C2B—C3B	179.69 (16)
O44A—S41A—C41A—C51A	38.29 (16)	C2B—C1B—C1B^i^—C2B^i^	180.00 (17)
O45A—S41A—C41A—C31A	−22.51 (16)	C1B^i^—C1B—C6B—C5B	−179.65 (19)
O45A—S41A—C41A—C51A	159.56 (14)	C6B—C1B—C2B—C3B	0.0 (3)
O46A—S41A—C41A—C31A	95.98 (15)	C1B—C2B—C3B—C4B	0.8 (3)
O46A—S41A—C41A—C51A	−81.95 (15)	C2B—C3B—C4B—S4B	176.90 (14)
O43B—S4B—C4B—C5B	−24.14 (18)	C2B—C3B—C4B—C5B	−1.5 (3)
O42B—S4B—C4B—C3B	35.67 (16)	C3B—C4B—C5B—C6B	1.6 (3)
O41B—S4B—C4B—C3B	−82.09 (15)	S4B—C4B—C5B—C6B	−176.87 (17)
O41B—S4B—C4B—C5B	96.31 (16)	C4B—C5B—C6B—C1B	−0.9 (3)
O42B—S4B—C4B—C5B	−145.93 (16)	N1C—C2C—C3C—C4C	50.99 (19)
O43B—S4B—C4B—C3B	157.47 (15)	C2C—C3C—C4C—C41C	−174.92 (14)
C6C—N1C—C2C—C3C	−55.64 (19)	C2C—C3C—C4C—C5C	−51.75 (19)
C2C—N1C—C6C—C5C	60.94 (19)	C3C—C4C—C41C—N41C	−71.7 (2)
C6D—N1D—C2D—C3D	60.39 (18)	C3C—C4C—C41C—O41C	107.02 (19)
C2D—N1D—C6D—C5D	−59.47 (18)	C5C—C4C—C41C—N41C	166.30 (18)
C2E—N1E—C6E—C5E	−59.72 (18)	C41C—C4C—C5C—C6C	179.63 (14)
C6E—N1E—C2E—C3E	59.69 (19)	C3C—C4C—C5C—C6C	57.26 (18)
C2A—C1A—C11A—C61A	−143.92 (19)	C5C—C4C—C41C—O41C	−15.0 (2)
C11A—C1A—C6A—C5A	176.71 (16)	C4C—C5C—C6C—N1C	−61.90 (18)
C6A—C1A—C2A—C3A	2.2 (3)	N1D—C2D—C3D—C4D	−57.77 (17)
C2A—C1A—C6A—C5A	−1.5 (3)	C2D—C3D—C4D—C41D	175.04 (14)
C6A—C1A—C11A—C21A	−141.7 (2)	C2D—C3D—C4D—C5D	54.23 (19)
C6A—C1A—C11A—C61A	37.9 (2)	C3D—C4D—C41D—O41D	−12.3 (2)
C11A—C1A—C2A—C3A	−176.04 (19)	C3D—C4D—C41D—N41D	169.59 (18)
C2A—C1A—C11A—C21A	36.5 (3)	C5D—C4D—C41D—N41D	−69.2 (2)
C1A—C2A—C3A—C4A	−0.9 (3)	C5D—C4D—C41D—O41D	108.90 (19)
C2A—C3A—C4A—S4A	174.89 (17)	C3D—C4D—C5D—C6D	−53.70 (19)
C2A—C3A—C4A—C5A	−1.2 (3)	C41D—C4D—C5D—C6D	−175.91 (14)
C3A—C4A—C5A—C6A	1.8 (3)	C4D—C5D—C6D—N1D	56.29 (18)
S4A—C4A—C5A—C6A	−174.16 (13)	N1E—C2E—C3E—C4E	−57.49 (18)
C4A—C5A—C6A—C1A	−0.5 (3)	C2E—C3E—C4E—C5E	54.81 (19)
C1A—C11A—C21A—C31A	−179.60 (18)	C2E—C3E—C4E—C41E	175.84 (14)
C21A—C11A—C61A—C51A	−0.8 (3)	C41E—C4E—C5E—C6E	−175.66 (14)
C1A—C11A—C61A—C51A	179.60 (16)	C3E—C4E—C41E—O41E	−20.9 (2)
C61A—C11A—C21A—C31A	0.8 (3)	C3E—C4E—C41E—N41E	161.03 (18)
C11A—C21A—C31A—C41A	−0.7 (3)	C5E—C4E—C41E—O41E	100.16 (19)
C21A—C31A—C41A—C51A	0.6 (3)	C5E—C4E—C41E—N41E	−77.9 (2)
C21A—C31A—C41A—S41A	−177.32 (16)	C3E—C4E—C5E—C6E	−54.01 (19)
C31A—C41A—C51A—C61A	−0.6 (3)	C4E—C5E—C6E—N1E	56.66 (18)

Symmetry codes: (i) −*x*+2, −*y*+1, −*z*+1.

### Hydrogen-bond geometry (Å, °)

**Table:**

*D*—H···*A*	*D*—H	H···*A*	*D*···*A*	*D*—H···*A*
N1C—H11C···O42A^i^	0.91 (2)	1.99 (2)	2.889 (2)	169 (2)
N1C—H12C···O45A^ii^	0.90 (3)	1.95 (3)	2.838 (2)	168.1 (18)
N1D—H11D···O43A	0.90 (2)	2.04 (2)	2.878 (2)	154.6 (16)
N1D—H11D···O43B	0.90 (2)	2.407 (18)	2.855 (2)	111.0 (15)
N1D—H12D···O46A^iii^	0.91 (2)	1.98 (2)	2.877 (2)	170 (2)
N1E—H11E···O42B	0.901 (19)	2.003 (19)	2.878 (2)	163.5 (18)
N1E—H12E···O44A^iv^	0.93 (2)	2.508 (18)	2.908 (2)	106.2 (15)
N41C—H42C···O41E	0.89 (3)	1.95 (3)	2.836 (3)	178 (2)
N41D—H41D···O41D^v^	0.94 (3)	2.00 (3)	2.936 (3)	171 (2)
N41D—H42D···O41C^vi^	0.85 (3)	2.34 (3)	3.134 (2)	158 (2)
N41E—H41E···O41D^vii^	0.89 (3)	2.16 (3)	2.996 (2)	158 (3)
N41E—H42E···O41C	0.89 (3)	2.13 (3)	3.014 (3)	172 (2)
C5B—H5B···O43B	0.93	2.57	2.939 (3)	104
C2C—H21C···O42B^viii^	0.97	2.36	3.276 (2)	156
C2D—H21D···O41A^ix^	0.97	2.57	3.423 (2)	147
C31A—H31A···O45A	0.93	2.58	2.944 (2)	104
C51A—H51A···O41B^i^	0.93	2.45	3.367 (2)	169
C6C—H61C···O41A^x^	0.97	2.41	3.295 (2)	152
C6E—H61E···O44A^i^	0.97	2.46	3.345 (2)	151
C6D—H62D···O41A^ix^	0.97	2.52	3.380 (2)	148

Symmetry codes: (i) −*x*+2, −*y*+1, −*z*+1; (ii) −*x*+2, −*y*+2, −*z*+1; (iii) *x*, *y*−1, *z*; (iv) *x*−1, *y*−1, *z*; (v) −*x*+1, −*y*−1, −*z*; (vi) *x*, *y*−1, *z*−1; (vii) −*x*+1, −*y*, −*z*+1; (viii) −*x*+1, −*y*+1, −*z*+1; (ix) −*x*+1, −*y*, −*z*; (x) *x*, *y*+1, *z*+1.
